# Characterization of the Viable but Nonculturable (VBNC) State in *Saccharomyces cerevisiae*

**DOI:** 10.1371/journal.pone.0077600

**Published:** 2013-10-29

**Authors:** Mohammad Salma, Sandrine Rousseaux, Anabelle Sequeira-Le Grand, Benoit Divol, Hervé Alexandre

**Affiliations:** 1 UMR PAM Université de Bourgogne-AgroSup Dijon Laboratoire VALMIS Institut Universitaire de la Vigne et du Vin Jules Guyot, Université de Bourgogne, Dijon, France; 2 Institute for Wine Biotechnology, Stellenbosch University, Stellenbosch, South Africa; 3 Plateforme de Cytométrie, Université de Bourgogne, Dijon, France; University of Florida, United States of America

## Abstract

The Viable But Non Culturable (VBNC) state has been thoroughly studied in bacteria. In contrast, it has received much less attention in other microorganisms. However, it has been suggested that various yeast species occurring in wine may enter in VBNC following sulfite stress.In order to provide conclusive evidences for the existence of a VBNC state in yeast, the ability of *Saccharomyces cerevisiae* to enter into a VBNC state by applying sulfite stress was investigated. Viable populations were monitored by flow cytometry while culturable populations were followed by plating on culture medium. Twenty-four hours after the application of the stress, the comparison between the culturable population and the viable population demonstrated the presence of viable cells that were non culturable. In addition, removal of the stress by increasing the pH of the medium at different time intervals into the VBNC state allowed the VBNC *S. cerevisiae* cells to “resuscitate”. The similarity between the cell cycle profiles of VBNC cells and cells exiting the VBNC state together with the generation rate of cells exiting VBNC state demonstrated the absence of cellular multiplication during the exit from the VBNC state. This provides evidence of a true VBNC state. To get further insight into the molecular mechanism pertaining to the VBNC state, we studied the involvement of the *SSU1* gene, encoding a sulfite pump in *S. cerevisiae*. The physiological behavior of wild-type *S. cerevisiae* was compared to those of a recombinant strain overexpressing *SSU1* and null *Δssu1* mutant. Our results demonstrated that the *SSU1* gene is only implicated in the first stages of sulfite resistance but not per se in the VBNC phenotype. Our study clearly demonstrated the existence of an SO_2_-induced VBNC state in *S. cerevisiae* and that the stress removal allows the “resuscitation” of VBNC cells during the VBNC state.

## Introduction

Microorganisms, like all living organisms, naturally respond to changing environmental conditions. They display a remarkable ability to adapt to certain physical and chemical stresses in their environment. Survival mechanisms are activated following the detection of environmental signals and generate a complex adaptive response that leads to a state of tolerance and thus survival under sub-optimal or even sub-lethal conditions [1]. When the environmental conditions threaten their survival or prevent them from living in optimal conditions, the cells are described as stressed [2]. This notion of stress plays a fundamental role in the survival of microorganisms in foodstuff. Giraffa et al. [3] argued that the ability of microorganisms to grow, survive and display a metabolic activity in foodstuffs is the result of stress response.

However, between the unstressed state and death, different physiological states have been described: viable and culturable, injured, dormant, viable but non culturable (VBNC) and dead [4]. These physiological adaptations require a variable response time depending on the intensity and abruptness of exposure to the stress-inducing factor(s). The VBNC state, which has been extensively studied in bacteria, is characterized by an inability of the cells to grow on culture media, even though they are still viable and maintain a detectable metabolic activity [5]. This state is reversible upon return of favorable conditions. Various environmental factors can induce entry into VBNC state: temperature [6], [7], the physiological age of the culture, salinity [8], the oxygen content [9], light and ventilation [10]. Most studies on VBNC cells have focused on pathogenic bacteria. More than 60 bacterial species are described as being able to enter into a VBNC state, Gram-positive *(e.g. Listeria monocytogenes, Enterococcus, Micrococcus luteus*) and Gram negative *(e.g. Escherichia coli, Vibrio cholerae, Vibrio vulnificus, Legionella pneumophila, Campylobacter jejuni, Salmonella enterica, Pseudomonas aeruginosa, Helicobacter pylorii*) [11]. In contrast, the VBNC state has received much less attention in other microorganisms.

The existence of a VBNC state comparable to that described in bacteria has been suggested for the yeast *Saccharomyces cerevisiae* [12], [13]. A loss of culturability but not of viability has indeed been reported following an electrolytic low amperage shock and suggesting a physiological state comparable to the bacterial VBNC state [14]. Similarly, Bleve *et al.* [15] detected the presence of *S. cerevisiae* in a VBNC-like state in pasteurized foodstuffs. In addition, an ecology study conducted during alcoholic fermentation of sweet wines, suggested the presence of cells in a VBNC state in *Candida stellata* [16]. Sulfur dioxide (SO_2_) has been identified as the chemical stress factor inducing VBNC state in *Brettanomyces bruxellensis* grown in a wine synthetic medium [17]–[19]. The same observations were made for *S. cerevisiae* and *Zygosaccharomyces bailii* [20]. In order to sustain the hypothesis that the VBNC state is a physiological survival mechanism, it ultimately requires demonstrating the recovery of the culturable state from a VBNC population cells after removal of the stress factor [11], [21]. This resuscitation process is often triggered simply by removal of the stress that initially induced the VBNC response [11], [18]. There has also been numerous reports of resuscitation induced by other mechanisms such as nutrient addition [22], temperature upshift [23] and heat shock [24]. However, most of these resuscitation processes were successful only with cultures which had been in the VBNC state for only a short period of time [17], [21], [25]. In the case of wine yeasts that entered into a VBNC state as a response to SO_2_ exposure, various authors have shown that a substantial decrease in molecular SO_2_ concentration induced resuscitation [17], [19], [20].

A number of methods have been employed to examine the viability of non culturable cells in order to suit different needs. The viability of bacteria can indeed be assessed in populations (bulk assay) or in single cells (cytological assay) [4], [25]. The latter appear to be preferred since it is based on growth-independent viability techniques such as the assessment of cell viability by the maintenance of stable cellular structure. These methods include the use of nucleic acid stains, redox indicators, membrane potential probes or metabolic indicators such as fluorescein diacetate that can be detected by fluorescence microscopy [19], [20] or flow cytometry [17].

In this study, we evaluated the effect of SO_2_ on the entry of *S. cerevisiae* cells into the VBNC state, the resuscitation capability of VBNC *S. cerevisiae* cells using a flow cytometry and trying to demonstrate that the recovery of culturability is due to a true resuscitation and not to the presence and growth of a few residual cells with a normal metabolism. Finally, we investigated the role of *SSU1* in the VBNC state.

## Materials and Methods

### Strains, Plasmids and Culture Conditions

The different bacteria, yeast strains and plasmid used in this study are listed in Table 1.

**Table 1:** Yeast strains used in this study.

Strains and plasmid	Genotype/Description	Reference
*S. cerevisiae* S288C	*MATα SUC2 gal2 mal mel flo1 flo8-1* Δ *hap1*	
*S. cerevisiae* BYD4742	S288C derivative, *MATα his3*Δ*1 leu2*Δ*0 lys2*Δ*0 ura3*Δ*0*	[46]
*S. cerevisiae* BYD4742 *Δssu1*	BYD4742 derivative, *MATα his3*Δ*1 leu2*Δ*0 lys2*Δ*0* *ura3*Δ*0 ssu1::KanMX4*	EUROSCARF deletion library*
*Escherichia coli* DH5α	[*F^−^φ80lacZΔM15Δ(lacZYAargF) U169 deoR recA1 endA1* *hsdR17(rk−, mk+) phoA supE44 thi-1gyrA96 relA1 λ*]	GIBCO-Invitrogen Life technologies, Mowbray,South Africa
pCEL13	*2 µm Ap^R^ URA3 PGK1_P_–PGK1_T_*	[27]
pCEL13-*SSU1*	*2 µm Ap^R^ URA3 PGK1_P_-SSU1-PGK1_T_*	This study

* http://www.uni-frankfurt.de/fb15/mikro/euroscarf/yeast.html.

Plasmids were constructed and amplified in *Escherichia coli* DH5α, grown in Luria Bertani (LB) medium (Biolab diagnostics, Wadenville, South Africa). The medium was supplemented with 100 mg/L ampicillin for the selection of resistant bacteria when appropriate. *S. cerevisiae* (S288C, BYD4742 and BYD4742Δ*ssu1*) strains were grown in Yeast Peptone Dextrose (YPD) (10 g/L yeast extract, 10 g/L Bacto-peptone, 20 g/L glucose) at 28°C. For the selection of yeast transformants, Synthetic Complete (SC) medium containing 20 g/L glucose, 6.7 g/L yeast nitrogen base with ammonium sulfate and amino acids (Difco Scientific group, Waterfall Park, South Africa) supplemented with 60 µg/mL leucine and 30 µg/mL lysine to apply a uracil auxotroph.

### DNA Preparation and Analysis

Chromosomal DNA from *S. cerevisiae* BYD4742 strain was isolated from overnight culture grown in YPD at 30°C [26]. The *SSU1* gene was amplified by polymerase chain reaction (PCR) using the 5′ScSSU1fw (GGATCCATGGTTGCCAATTGGGTACTT) and 3′ScSSU1rev (CTCGAGTTATGCTAAACGCGTAAAATCTAGAG) primers in an Applied Biosystems 2720 thermal cycler. Phusion DNA polymerase enzyme (Finnzymes, Finland) and Phusion buffer (Finnzymes, Thermo Scientific, Pretoria, South Africa) with MgCl_2_ were used. The reaction mixture contained Phusion DNA polymerase enzyme (1 U), Phusion buffer (1X), 250 µM of each nucleotide (dNTP), 200 ng genomic DNA, 0.25 µM of each primer, and 0.2 mM MgCl_2_. The PCR program is consisted of a 30 s initial denaturation cycle at 98°C (initial denaturation), followed by 35 cycles of 98°C for 10 s (denaturation), 58°C for 45 s (annealing), 72°C for 50 s (elongation). The program ended with a final 10 min extension at 72°C (final elongation).

The amplicons obtained were cloned into a pJET1.2 using the CloneJet PCR Cloning Kit (Thermo Scientific) according to the manufacturer’s instructions. Plasmid DNA was isolated from positive transformants of *E. coli* DH5α using the Qiaprep Spin Miniprep Kit (Qiagen, Whitehead Scientific, Cape Town, South Africa). Both strands were sequenced in an ABI 3130XL Genetic Analyzer at the Central Analytical Facility (Stellenbosch University) using the pJET1.2 Forward and Reverse sequencing primers.

### Constructing Overexpression Vectors

The *SSU1* gene was then subcloned into the pCEL13 yeast expression vector [27] (Table 1) as follows: *SSU1* was excised from pJET1.2 restriction with *Bam*HI and *Xho*I (Roche Diagnostics, Randburg, South Africa) and ligated into the *Bgl*II and *Xho*I sites of the pCEL13 expression vector respectively to yield a plasmid named pCEL13-*SSU1*. Restriction endonuclease-digested DNA was eluted from agarose gels by using the ZymocleanTM gel recovery kit (Zymo research, USA) according to the manufacturer’s instructions. Standard methods were used for the restriction and ligation of DNA, plasmid transformation into *E. coli*, and agarose-gel electrophoresis [28].

### Yeast Transformation

*S. cerevisiae,* strain BYD4742Δ*ssu1* was transformed with pCEL13-*SSU1*. Yeast transformation was conducted using an electroporation method as previously described [29]. The plasmids were maintained as autonomously replicating plasmids in the yeast cells by growing yeast cells cultured in uracil deficient media. Transformation was verified by colony PCR analysis using the 5′KPNPGK-631(GGGGTACCCTTTATTTTGGCTTCACCC) and 3′PGKKPN-1378 (CGCGGGGGTACCGATAAATAATAGTCTATATATACG) primers. The reaction was performed in 50 µl using 1×*Taq* buffer (Promega Corp., USA), 250 µM of each nucleotide dNTPs, 1.5 mM MgCl_2_, and 1 unit of *Taq* DNA polymerase (Promega Corp., USA) with the following cycling conditions: 10 min initial denaturation cycle at 98°C (initial denaturation), followed by 30 cycles of 98°C for 10 s (denaturation), 58°C for 30 s (annealing), 72°C for 50 s (elongation). The program ended with a final 10 min extension at 72°C (final elongation). PCR products were resolved on 1% agarose gel prepared with 1×TBE buffer and 1 µM of ethidium bromide and visualized under UV-light; the relative molecular length of the PCR product was estimate to be about 2 Kpb in order to validate the yeast transformation (results not shown).

### Adaptation of Different Strains to the Synthetic Wine Medium

*S. cerevisiae* S228C, BYD4742 and BYD4742Δ*ssu1*strains were grown on YPD, *S. cerevisiae* BYD4742Δ*ssu1*pCEL13-*SSU1* was grown on SC at 28°C, for 5 days as starter inocula.

For *S. cerevisiae* S228C,VBNC studies were performed in synthetic wine (SW) (8% ethanol, 3 g/L D-L malic acid, 0.01% acetic acid, 0.1 g/L potassium sulfate, 0.025 g/L magnesium sulfate, 1 g/L yeast extract, 1.5 g/L glucose, 1.5 g/L fructose). VBNC studies for *S. cerevisiae* BYD4742, BYD4742Δ*ssu1* and BYD4742Δ*ssu1* pCEL13-*SSU1* was performed in modified synthetic wine (MSW) (8% ethanol, 3 g/L D-L malic acid, 0.01% acetic acid, 0.1 g/L potassium sulfate, 0.025 g/L magnesium sulfate, 1.5 g/L glucose, 1.5 g/L fructose, 6.7 g/L yeast nitrogen base with ammonium sulfate and amino acids supplemented with 60 µg/mL leucine and 30 µg/mL lysine) supplemented with 50 µg/mL uracil for the culture of *S. cerevisiae* BYD4742, BYD4742Δ*ssu1* strains.

The pH was adjusted to 3.5, using 2 M NaOH and the medium was filter-sterilized using 0.2 µm filters (Millipore, Molsheim, France). One single colony was inoculated into 10 mL of SW-YPD (50∶50) (*S. cerevisiae* S228C) or in 10 mL of MSW-SC (50∶50) (*S. cerevisiae* BYD4742, BYD4742Δ*ssu1*, and BYD4742Δ*ssu1* pCEL13 *SSU1*) and incubated at 28°C for 3 days. 5.10^5^ cell/mL from this preculture were inoculated into 1 L SW or MSW, depending on the strain, and incubated at 28°C for 3 days in order to obtain approximately 10^7^ cell/mL.

### Culturability and Viability Assays

Samples of *S. cerevisiae* suspensions were taken at various time points during incubation at 28°C in SW or MSW, for the determination of total, viable and culturable populations. Cell culturability was assessed by a spread plating procedure on YPD agar or SC agar depending on the strain. The percentage of cells that were viable was expressed as total cell counts determined by flow cytometry (FCM). Two fluorescent dyes, namely fluorescein diacetate (FDA) and FUN-1, were used to evaluate the viability of *S. cerevisiae* using FCM. FDA is a lipophilic, uncharged and non-fluorescent substrate for cellular esterase that cleaves FDA inside living cells to release green fluorescent fluorescein (emission at 520 nm). FDA is therefore used to monitor cellular esterase activity and to determine the viability of cell populations. For the staining procedure, 0.5 mL of cultured cells was added to 0.5 mL of FDA buffer ((0.5 M Na_2_HPO_4_ (Sigma 255793, France; pH 7.4) and 0.5 M NaH_2_PO_4_(Sigma S2554, France); pH 7)) to which 1.5 µL of FDA at 10 µM in acetone (Sigma F737, USA) was added in order to reach a final concentration of 15 µM, and the cells were then incubated for 15 min at room temperature in the dark before being analyzed by FCM.

Furthermore, another viability probe (FUN-1) (Invitrogen F-7030) was used in order to validate the presence of the metabolic activity in the non culturable cells. FUN-1 [2 chloro 4 (2,3 dihydro 3 methyl (benzo 1,3 thiazol-2-yl) methylidene) 1 phenylquinolinium iodide] is a fluorescent probe that belongs to a class of halogenated asymmetric cyanine dyes and is essentially non-fluorescent in aqueous solution. FUN-1 stains nucleic acids, producing a green to green-yellow fluorescence in membrane compromised dead yeast cells [30]. In metabolically active yeast, cylindrical intravacuolar structures (CIVS) are produced after less than 1 h exposure to FUN-1 [30]. This stain gives rise to the formation of CIVS structures in the vacuoles of metabolically active yeast cells grown and stained under either oxidative or fermentative conditions [30]. These structures often appear to move within a vacuolar space and are red when excited at 470–590 nm. To stain cells with FUN-1 different suspensions of live, dead, and non culturable yeast cells were analyzed. *Saccharomyces cerevisiae* S288C cells were washed in sterile PBS (130 mM NaCl (Sigma-Aldrich #S9888, St Quentin Fallavier, France) 5 mM NaH_2_PO_4_ (Sigma-Aldrich #S2554) and 5 mM Na_2_HPO_4_ (Sigma-Aldrich #255793) pH 7.2) and a portion was killed using Natamycin (yeast cells treated with Natamycin (Delvocid) (Humeau, France), for 60 min at 28°C). The absence of viability was confirmed by absence of growth on YPD agar media and by FCM analysis using FUN-1. Live, dead and non culturable yeasts were stained separately using FUN-1, 1 mL of each *S. cerevisiae* suspension was washed twice with PG solution (PBS pH 7.2 containing 2% glucose). A centrifugation at 10000 g for 5 min was performed and the pellet was resuspended in PG solution (this solution ensures that yeasts remain metabolically active during the experiment). The cells were then incubated with FUN-1 at a final concentration of 15 µM for 30 min at 28°C. Cells were analyzed by FCM. A dot plot of Red fluorescence (*y*-axis) over Green fluorescence (*x*-axis) was prepared.

### Flow Cytometry Analysis

FCM samples were analyzed using a Guava EasyCyte Plus SSC4C flow cytometer (Guava Technologies, Hayward). This instrument is equipped with a 488-nm, 25-mW laser line, forward scatter (FSC, for cell size) and side scatter (SSC, for granularity) detectors; green fluorescence was collected on the FL 1 channel using a 525-nm (±30 nm) band-pass filter red fluorescence was collected on the FL 3 channel using a 680-nm (±30 nm) band-pass filter. This instrument allows determining accurate cell numbers and population percentages, without the need for reference beads, as described by the manufacturer using only the Guava Cytosoft data acquisition and analysis software. For all analyses, a minimum of 5,000 events was acquired, and all samples were collected as logarithmic signal. Experiments were performed in duplicate and included an unlabeled sample as a control in 96-well plates. Data were analyzed using the Guava Cytosoft data acquisition and analysis software version 5.0 and FlowJo software version 7.6.

### Induction of Entry into and Exit from the VBNC State

Based on studies that have been carried out previously [17], [20], SO_2_ was used to induce the VBNC state. In wine, different species of SO_2_ are in a pH-dependent equilibrium: HSO_3_^−^, SO_3_^2−^ and molecular SO_2._ The latter is the main antimicrobial species of SO_2_ [31]. When pH decreases, the concentration of molecular SO_2_ increases as does the antimicrobial strength for a given total SO_2_ concentration [31].

Entry into the VBNC state was induced by adding different concentrations of molecular SO_2_ (ranging between 0.1 mg/L and 4.5 mg/L). Desired molecular SO_2_ concentration was obtained using potassium metabisulfite solution. The level of potassium metabisulfite to be added was determined as reported previously [32] taking into account, the pH of the medium and the pKa of SO_2_. Exit from the VBNC state was induced at different time intervals after addition of SO_2_ (i.e. 3, 7, 14, 21, and 30 days) by adjusting the pH to 4.0 via the addition of 2 M NaOH. A pH of 4.0 was indeed found sufficient to bring the concentration of molecular SO_2_ close to 0. All of our studies of entry into and exit from the VBNC state were performed in triplicate. The percentage of viable cells was calculated as follows: % viability = (viable cell count/total cell count)×100.

### FDA Reliability Assay

A synthetic wine was inoculated with *S. cerevisiae* S288C strain to a final concentration of 5.10^5^ CFU/mL and incubated for 3 days at 28°C to obtain approximately 10^7^ CFU/mL. Thereafter, different lethal stresses such as Natamycin (25 mg/L) and SO_2_ (10 g/L) were applied. Every 5 min 1 mL of cells was centrifuged (13,000 g for 5 min at 25°C), the pellet was rinsed twice in PBS and cell culturability was assessed by a spread plating procedure on YPD media. Green fluorescent intensity was determined by flow cytometry using FDA.

### VBNC Cell Cycle Analysis

A comparison of the cell cycle profiles of cells in VBNC state and cells exiting the VBNC state was carried out in order to show the absence of cell proliferation during the exit from the VBNC state using FCM and propidium iodide (PI), a red fluorescent probe (635 nm emission) that binds to the nucleic acid [33], [34]. 1 mL of cell suspension of *S. cerevisiae* S288C from exponentially growing culture in SW (control), VBNC and culturable cells exiting from the VBNC state were centrifuged for 5 min at 10,000 g, the pellet was suspended in 1 mL cold 70% ethanol and the tubes were stored for 3 hours at 4°C. Cells were suspended in 1 mL 50 mM citrate buffer pH 7 (Sigma-Aldrich #S4641) after 5 min centrifugation at 10,000 g. A second centrifugation for 5 minutes at 10,000 g was performed and the cells were suspended in 1 mL 50 mM citrate buffer pH 7 containing 0.25 mg/mL RNase A (Sigma-Aldrich #R4875), to ensure DNA-specific binding as PI can stain both double-stranded RNA and DNA [35]. Incubation for 1 h at 50°C was then carried out. In order to stain cells with PI, the tubes were centrifuged again for 5 min at 10,000 g and the pellet was resuspended in citrate buffer pH 7 containing 8 µg/mL PI (Sigma-Aldrich #81845) and stored at 4°C for 3 days. All analyses were carried out in triplicate at a concentration of 10^6^ cell/mL. PI is detected in the 575/26 nm channels on the BD LSRII which is equipped with a 488-nm, 22-mW laser line, forward scatter (FSC) and side scatter (SSC) detectors. Initial cell population gating is placed on FSC vs SSC (cell size vs granularity). This cell population gate was then placed on PE 575/26 nm-W(width) vs PE 575/26 nm-A(Area) plot. Doublets appear to the right of single cell analysis (gate P2). Single cell gate P2 was then displayed as a histogram using PE 575/26 nm-A parameter. For all analyses, a minimum of 10,000 events was acquired, and all samples were collected as linear signal. Cell Cycle analysis of research samples was adequately done using the FlowJo software.

### VBNC Exit Rate Assay

A comparison of the cell generation time and exit rate of the VBNC cells was carried out in order to further verify the absence of cell proliferation during the exit from the VBNC state. A filtered SW pH 4.0 obtained from a culture of *S. cerevisiae* S288C (14 days, in synthetic wine containing 8% ethanol) was inoculated with the same strain to a final concentration of 10^4^ CFU/mL. This culture was used to determine the generation time (doubling time of the biomass in the exponential phase) of *S. cerevisiae* S288C, under the same experimental conditions, during the exit from the VBNC state. To determine the generation time (G), the optical density at 600 nm as well as plate counting on YPD agar were determined every 2 h and compared to the exit rate of the VBNC state which was determined after the removal of the SO_2_ stress as described above. The generation time and the exit rate were calculated according to the following formula: G = ln(2)/μ (max) wih ln(N2)-ln(N1) = μ (max) (t2-t1), (N2 is cell number at t2 and N1 is the cell number at t1).

## Results and Discussion

### Evidence for a VBNC State in *S. cerevisiae* (Induction and Exit)

**Figure 1:**
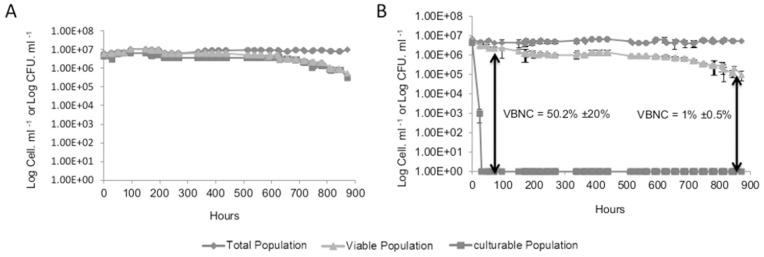
Changes in the total cell population (♦) culturable population (▪), and viable population (▴) of a culture of *S. cerevisiae* S288C on incubation at 28°C. Panel A shows the growth control condition in synthetic wine. Panel B shows the induction of VBNC state in *S. cerevisiae* S288C in synthetic wine with the addition of 4.5 mg/L molecular SO_2_ at time 0. Value 1 corresponds to an undetectable number (less to 10 CFU/mL).The values presented are the average of three replicates of three separate experiments.

SO_2_ was used as a stress factor in an attempt to induce the VBNC state in *S. cerevisiae*. FCM counts of total or viable cells using FDA and culturable cell counts were compared in order to monitor the entry of *S. cerevisiae* S288C cells into the VBNC state. In the absence of SO_2_, more than 95% of total cells remained viable and cultivable during the first three days (Fig. 1A). Entry into the VBNC state was assayed by incubation of the cells with different concentrations of molecular SO_2_, ranging from 0.1 to 4.5 mg/L. When 4.5 mg/L of molecular SO_2_ were added 3 days after synthetic wine was inoculated (Time 0), the viability and the culturability of cells decreased rapidly and all viable cells became non culturable after 48 h (Fig. 1B). When applying lower concentrations of SO_2_, some viable cells always remained culturable (data not shown). In the first 3 days following the addition of SO_2_, a decrease of viability from 4.2×10^6^ to 2.2×10^6^ cells/mL was observed and could be explained by the fact that some cells are more sensitive to SO_2_ than others. In the third day (72 h) following the sulfite stress, no more colonies were detected on YPD medium. The difference between the percentage of culturable cells and viable cells suggests that a significant proportion of cells were in a VBNC state (Fig. 1B). For strain S288C, 52%±20% (2.2×10^6^ cells/mL)in average of the total population was in VBNC state after 3 days and 1%±0,5% (4.2×10^4^ cells/mL) remained in a VBNC state 36 days after stress exposure while the rest of the population died.

**Figure 2:**
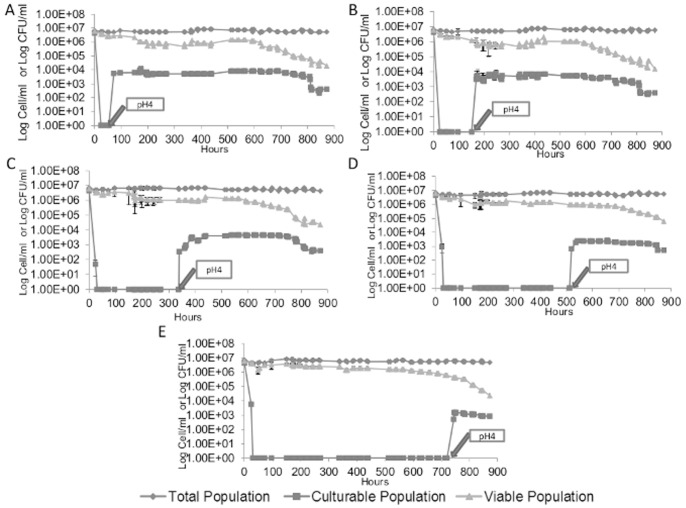
Resuscitation of *S. cerevisiae* S288C from the VBNC state. Total cell counts (♦), culturable counts (▪), and viable counts (▴) are shown. Resuscitation was induced by removal of the molecular SO_2_ at different time intervals after entry into VBNC state (i.e. A: 3 days, B: 7 days, C: 14 days; D: 21 days; E: 30 days). Value 1 corresponds to an undetectable number (less to 10 CFU/mL). The values presented are the average of three replicates of three separate experiments.

The ability of cells to exit from the VBNC state was investigated at different days (3, 7, 14, 21 and 29 days or 72 h, 168 h, 336 h, 504 h, 696 h respectively) (Fig. 2) by increasing the pH from 3.5 to 4.0 in order to decrease the molecular SO_2_ concentration [17]. In order to rule out the effect of pH on VBNC state, the effect of rising the pH on yeast growth dynamic has been checked (Fig. S1). It appears that pH increase did not lead to VBNC cells. One day after the pH-induced drop of the molecular SO_2_ concentration, approximately 1%±0,5% of the cells that initially entered into a VBNC state recovered culturability, regardless of the period of time they have been in the VBNC state (72 h, 168 h, 336 h, 504 h, 696 h ) (Fig. 2). The percentage of yeast recovering culturability started to decrease after few days depending on the time they were kept in VBNC state. It can be speculated that, all cells did not resuscitate because of the heterogeneity of physiological states within the yeast population [36].

In the current study, we managed to confirm the ability of *S. cerevisiae* to survive in a VBNC state over a long period of time (36 days, 864 h). The results indicate that *S. cerevisiae* becomes non culturable after three days in response to SO_2_ exposure but 52%±20% of the initial population remains viable as assessed by FDA probe (Fig. 1B). This observation agrees with the antimicrobial activity of SO_2_ [37] and with the hypothesis according to which SO_2_ induces a viable but non culturable state in *S. cerevisiae* [20]. Moreover, stress removal by increasing the pH of the growth medium allows VBNC cells to resuscitate (i.e. recover culturability) (Fig. 2).

### Metabolic Activity in Non-culturable Cells

**Figure 3:**
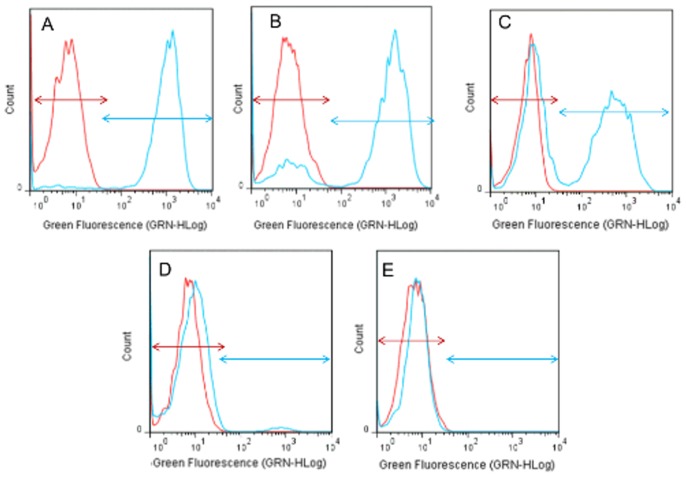
FCM histograms of *S. cerevisiae* S288C cells stained with FDA. The cells were incubated with 25/L natamycin in Synthetic wine at 28°C. After 30 (B); 60(C); 120(D) and 150(E) min, the cells were collected, and the cell Green fluorescence intensity was analyzed by FCM, Panel A represents control cells in the absence of SO_2_ (0 min). The Green fluorescence intensity (GRN-HLog) is represented on the x-axis, and cell counts are represented on the y-axis. Panels show the fluorescence of *S. cerevisiae* S288C before (red arrow; self-fluorescence) and after (blue arrow) staining with FDA. One representative experiment of the three performed is shown.

In order to ensure that the green fluorescence intensity observed in VBNC cells is a good reflection of metabolic activity and not a residual esterase activity, the green fluorescence intensity was measured in dead cells obtained using 2 lethal chemicals such as the exposure to natamycin (25 mg/L) or SO_2_ (10 g/L). Our results show that after 150 min of treatment with natamycin (25 mg/L) (Fig. 3) or 45 min of treatment with SO_2_ (10 g/L) (Fig. S2) no cells presented a green fluorescence. This indicates that the green fluorescence reflects a true metabolic activity and not a residual esterase activity. This validates that cells that are considered in VBNC state after being exposed to SO_2_ stress and still detectable by FCM (for a longer period of time more than 24 h after the loss of their culturability) are metabolically active in a VBNC state (Fig. 2).

**Figure 4:**
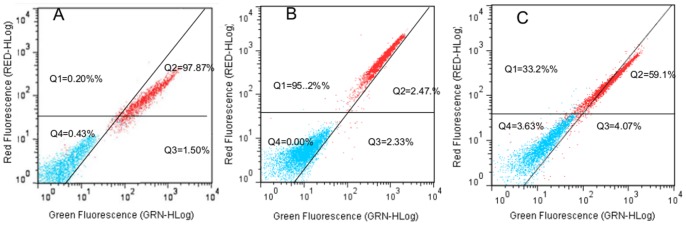
FCM analysis of *S. cerevisiae* S288C cells stained with FUN-1. Green fluorescence intensity is shown on the x-axes and red fluorescence intensity is shown on the y-axes. Dot plot (A) shows the fluorescence of dead cells after staining with FUN-1. Dot plot (B) shows the fluorescence of viable and culturable cells after staining with FUN-1. Dot plot (C) shows the fluorescence of VBNC cells after staining with FUN-1. Red-negative cells are contained in quadrants 3 and 4; Red-positive cells are contained in quadrants 1 and 2. Green- positive cells are contained in quadrants 2 and 3; Green-negative cells are contained in quadrants 1 and 4. Red fluorescence was measured at 630 nm (emission) and Green at 525 nm (emission). One representative experiment of the three performed is shown.

The analysis by FCM using FUN-1 of viable, dead and non cultrable (Viable and culturable cells treated with 4.5 mg/mL of SO_2_) cells of *S. cerevisiae* S288C was performed. The Green and the Red-labeled populations were spatially resolved in dot plots of FL1 and FL3. Analysis by FCM of the dead cells (treated with natamycin) stained by FUN-1 shows that more than 97.87% of cells diffused a green to green-yellow fluorescence indicating that the membrane was compromised as provided in a dead yeast cells (Fig. 4A). Analysis of viable (obtained 3 days after sulphite stress in synthetic wine) and culturable cells by FCM after staining by FUN-1 reveals the presence of a red fluoresence in 95.2% of the total population which indicates the formation of CIVS structures in the vacuoles of the metabolically active yeast cells (Fig. 4B).

33.2%±6% of the non culturable cells analyzed by FCM using FUN-1 displayed a red fluorescence (Fig. 4C). The presence of a red fluorecence in non culturable cells (33.2%±6%) showed that these cells present a CIVS structure that allows us to validate that a significant population within the non culturable cells present a metabolic activity.

### VBNC State Validation

**Figure 5:**
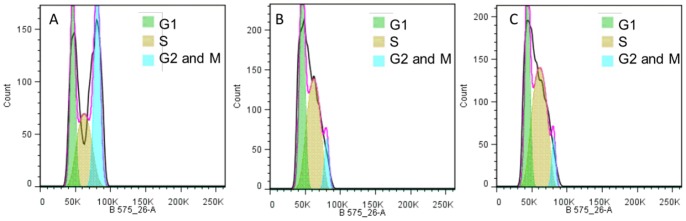
FCM analysis and cell cycle distribution of *S. cerevisiae* S288C. Analysis of *S. cerevisiae* S288C cell cycle during exponential phase in synthetic wine (Panel A), before (Panel B) and after exiting (Panel C) the VBNC state analyzed by FCM. The profiles Showed dual-variable plots of cell number versus PI uptake. G1 (green), S (brown), and G2/M (blue) cell populations were quantified. The experiment was repeated at least three times and representative data from single experiment is presented.

Proving the existence of the VBNC phenomenon as a physiological survival mechanism ultimately requires demonstrating the possible recovery of the culturable state from a non culturable population [11], [21]. Indeed, VBNC state can only be a significant means of survival if the cells surviving in this state are able to again recover their ability to multiply. In order to show that the recovery of culturability observed after the removal of the molecular SO_2_ stress (Fig. 2), is a true resuscitation and not a growth of a few residual viable and culturable cells with normal metabolism, a comparison of the profile of cell cycle in VBNC state just before and immediately after pH adjustment was performed using FCM. In addition, in order to determine the relative cellular DNA content, FCM was used to identify the cell distribution among the various phases of the cell cycle. The analysis of an exponentially growing population of *S. cerevisiae* S288C in synthetic wine medium using FCM with the DNA binding dye propidium iodide allowed the identification of the different phases of the cell cycle based on the theoretical distribution histogram of cells according to the linear relation between fluorescence intensity and by extrapolation the DNA content (Fig. 5A). 26.1% of the cells were detected with a 2C DNA content corresponding to the G1 phase (fluorescence intensity ≈ 45.103). 43.33% of the cells were detected with a 4C DNA content corresponding to the G2 and M phases (fluorescence intensity ≈ 90.103). Finally, 30.57% of the cells were found in the S phase, synthesizing DNA continuously and displaying a DNA content between 2C and 4C. The analysis of the cell cycle profile of cells in VBNC state (Fig. 5B) and cells exiting the VBNC state (3 days after the removal of the stress, VBNC percentage equal to 68%) (Fig. 5C), showed that most cells are in the S phase (43.3%) for both physiological states and exhibited similar profiles with an absence of a cell proliferation during resuscitation (Fig. 5A and B). Since the cell cycle profile is the same before and after exit from VBNC, this means that no cell multiplication occurred in the synthetic wine. This result together with the fact that after pH rising cells are culturable again (Fig. 2) demonstrated that these cells are able to again recover their ability to multiply.

**Figure 6:**
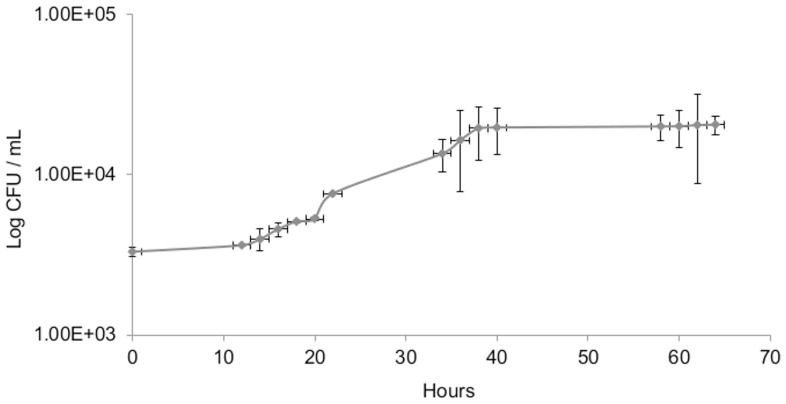
Growth of *S. cerevisiae* S288C in synthetic wine at 28°C. Error bars indicate the standard deviations of three independent experiments. The generation time is equal to 10 hours.

**Figure 7:**
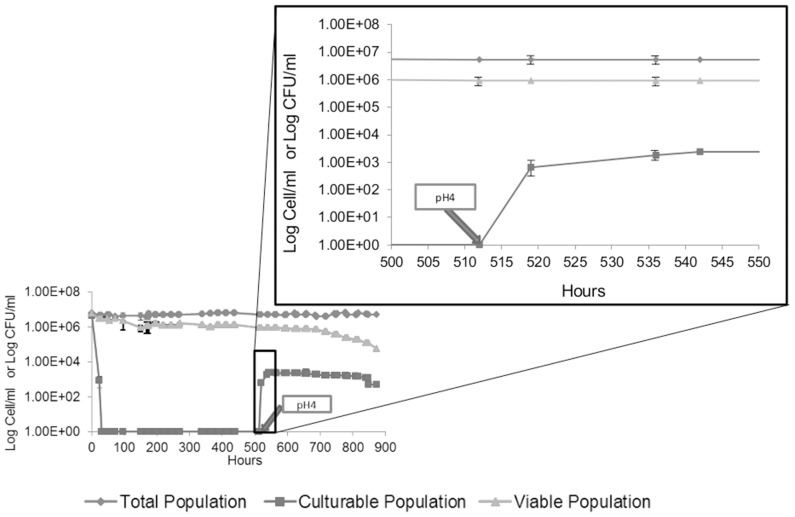
Exit rate of *S. cerevisiae* S288C from the VBNC. Total cell counts (♦), culturable counts (▪), and viable counts (▴) are shown. Resuscitation was induced by removal of the molecular SO_2_ at 21 days after entry into VBNC state. Value 1 corresponds to an undetectable number (less to 10 CFU/mL). The values presented are the average of three replicates of three separate experiments.

The generation time of *S. cerevisiae* S288C was determined by inoculating the S288C strain under the same experimental conditions during the exit from the VBNC state and was found to be approximately 10 h (Fig. 6). As, the culturability assay used in our study (100 µL on YPD agar) had a detection limit equivalent to 10 CFU/mL, consequently, during the resuscitation process, at least 56.9 h would have been required to reach a concentration of 5.19×10^2^ CFU/mL after the increasing of the pH, if the observed increase in culturability had been due to the presence of culturable cells. Yet our results show that 5.19×10^2^ CFU/mL of culturable cells were observed only 7 h after the pH increase (Fig. 7). According to the generation time calculated above, no viable and culturable cells would be able to grow up to 5.19×10^2^ CFU/mL in such a short period of time (7 h) (Fig. 7).

These results therefore validate the hypothesis of the VBNC state which is based on the fact that cells are able to regain their ability to multiply. This resuscitation has been strongly debated [21], [38], [39], as some authors suggest that the recovery of culturability is due to the presence and sudden growth of a few residual cells with a normal metabolism in a population predominantly non culturable. However, the recovery of cell division in a population of VBNC cells was described unambiguously for several bacteria [40], [41]. Cell resuscitation has been clearly demonstrated in vitro, in vivo and in situ [11]. In this study, the removal of environmental stress was sufficient to induce the exit from the VBNC state and the recovery in culturability observed was evidenced as a true resuscitation and not a simple growth of a few residual cells with a normal metabolism.

### Role of the Ssu1p Pump in the VBNC State

SO_2_ resistance mechanisms have been extensively studied in *S. cerevisiae*. SO_2_ detoxification, involving the plasma membrane protein Ssu1p, is one of the most efficient resistance mechanisms in this species [42]. Yeasts also tolerate SO_2_ by means of other systems, such as acetaldehyde production and the up-regulation of sulfite reduction systems [43]. The sulfite pump required for efficient sulfite efflux is encoded by the *SSU1* gene. Generally, mutations in *SSU1* cause sensitivity, whereas overexpression confers enhanced resistance to sulfite toxicity [44], [45].

**Figure 8:**
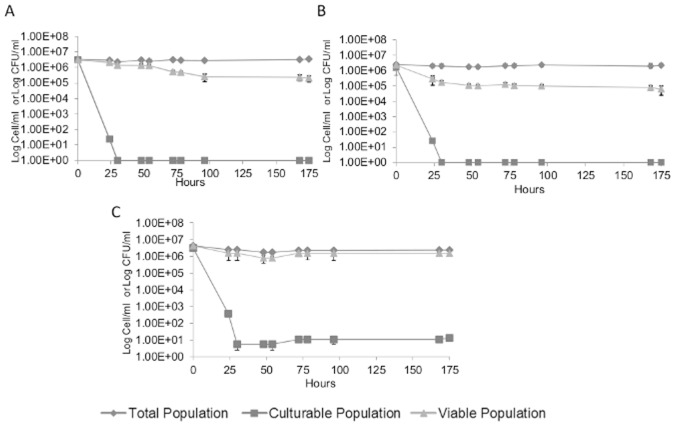
The induction of the VBNC state in *S. cerevisiae* BDY4742 (Panel A), BDY4742*Δssu1* (Panel B) and BDY4742Δ*ssu1* pCEL13 *SSU1* (Panel C) strain in synthetic wine with the addition of 4.5 mg.L^−1^ molecular SO_2_. Total cell counts (♦), culturable counts (▪), and viable counts (▴).Value 1 corresponds to an undetectable number (less to 10 CFU/mL). The values presented are the average of three replicates of three separate experiments.

In order to investigate the potential role of Ssu1p in the VBNC state of *S. cerevisiae,* the VBNC profiles of three strains, namely BYD4742, BYD4742*Δssu1* and BYD4742Δ*ssu1* pCEL13-*SSU1*, were compared. The study was carried out in a modified synthetic wine medium containing 4.5 mg/L of molecular SO_2_. Total and viable cell counts determined by flow cytometry and CFU counts (on SC agar) were compared in order to monitor the difference in the VBNC profile of the three strains. In the absence of SO_2_, more than 95% of total cells remain viable and cultivable during the first three days (data not shown). Entry into the VBNC state was induced by the addition of SO_2_ (4.5 mg/L molecular SO_2_) 3 days after synthetic wine inoculation (Time 0). The total population remained stable over time for all strains and culturability decreases quickly 30 h after SO_2_ addition to undetectable levels only for BYD4742 and BYD4742*Δssu1* (Fig. 8 A and B). However, for BYD4742Δ*ssu1* pCEL13-*SSU1*, some cells were still found culturable (5.5 cell/mL) even after 30 h of treatment (Fig. 8 C).

Moreover, the cell viability of BYD4742*Δssu1* decreased rapidly after the addition of SO_2_, to less than 10% 30 h after the treatment, whereas the viability of BYD4742 and BYD4742Δ*ssu1* pCEL13-*SSU1* strains decreased more slowly in the first few hours following the treatment (i.e. 60% of viability 30 h after treatment). This difference in the response to SO_2_ exposure between the wild-type, BYD4742Δ*ssu1* pCEL13-*SSU1* and BYD4742Δ*ssu1* allows to validate the role of *SSU1* in sulfite resistance mechanisms, as previously reported [42] and can be explained by the fact that the *SSU1* null mutant accumulated significantly more sulfite than the other strains (wild-type and BYD4742Δ*ssu1* pCEL13-*SSU1*), which make *SSU1* null mutant strain more sensitive to SO_2_.

**Figure 9:**
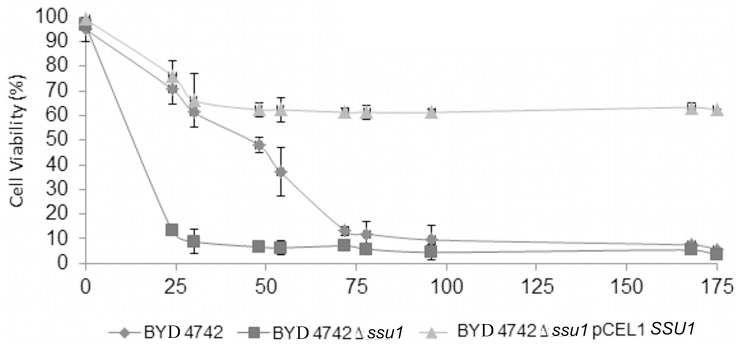
Viability percentage of *S. cerevisiae* BYD4742 (♦), BYD4742Δ*ssu1* (▪) and BYD4742Δ*ssu1* pCEL13 *SSU1*(▴) strain during the induction of the VBNC state. Viability percentage is determined by FCM using FDA. Error bars indicate the standard deviation of three independent experiments.

However, after 78 h, viability was no more significantly different and identical viability percentages (8%) were observed between the wild-type and BYD4742Δ*ssu1* (Fig. 8 A and B, Fig. 9) whereas 61% of BYD4742Δ*ssu1* pCEL13-*SSU1* cells remained viable. This could be explained by the fact that upon sudden exposure to a very high concentration of SO_2_ such as that required for entry into the VBNC state, the wild-type strain, unlike BYD4742Δ*ssu1* pCEL13-*SSU1*, does not have enough sulfite pumps in its membrane to efflux enough SO_2_ and detoxify the intracellular matrix. The overexpression of *SSU1* in a *SSU1* null mutant using the pCEL13 vector conferred enhanced resistance to sulfite toxicity as previously described [44], [45] ruling out the need for entry into a VBNC state (i.e. more than 99% of viable cells are non culturable). This result allows us to conclude that the *SSU1* gene is involved in sulfite resistance but not in the VBNC phenotype of *Saccharomyces cerevisiae*.

## Conclusion

*S. cerevisiae* S288C strain was used to generate conclusive evidence for the existence of a VBNC state in yeast, using a sulfite stress (4.5 mg/L molecular SO_2)_. For this purpose, cell count results obtained by FCM were compared to those obtained by plating on culture medium.

The addition of SO_2_ to a culture of *S. cerevisiae* induced entry into a VBNC state with a significant decrease of the metabolic activity. According to literature, the removal of the stressor factor can induce the exit from the VBNC state. In this study, the removal of molecular SO_2_ was performed by increasing the pH of the medium. Under these conditions, the ability of the cells to recover culturability after the stress removal was observed.

The green fluorescence detected by FCM using FDA reflected a true metabolic activity which indicates that cells that are considered in VBNC state after being exposed to SO_2_ stress and still detectable by FCM are metabolically active in a VBNC state (Fig. 2). This was further validated by the observation of CIVS structures, detected by FUN-1 probe. As the formation of these structures is strongly dependent on ATP, this further demonstrated the presence of metabolic activity.

We report that yeast cells can survive in a VBNC state in synthetic wine for up to one month. It is likely that *Saccharomyces* yeast cells could even stay longer in this state. The specific molecular mechanism involved in the entry into and exit from the VBNC state remains to be unraveled. A transcriptomic approach of VBNC cells would be useful to assess the existence of such a mechanism. From a practical point of view, this result demonstrates that the use of sulfite for stabilizing different beverages should be assessed using other methods than plating methods.

## Supporting Information

Figure S1 Effect of the increasing pH on the growth dynamic of a culture of *S. cerevisiae* S288C. Total cell counts (♦), culturable counts (▪), and viable counts (▴) are shown. pH increased at 3 days (72 h). The values presented are the average of three replicates of three separate experiments.
(TIFF)

Figure S2 FCM histograms of *S. cerevisiae* S288C cells stained with FDA. The cells were incubated with 10 g/L SO_2_ in Synthetic wine at 28°C. After 15 (B); 30(C); 45(D) and 60(E) min, the cells were collected, and the cell Green fluorescence intensity was analyzed by FCM, Panel A represents control cells in the absence of SO_2_ (0 min). The Green fluorescence intensity (GRN-HLog) is represented on the x-axis, and cell counts are represented on the y-axis. Panels show the fluorescence of *S. cerevisiae* S288C before (red arrow; self-fluorescence) and after (blue arrow) staining with FDA. One representative experiment of the three performed is shown.
(TIFF)
