# Supplementary figures and images for: Characterization of the Viable but Nonculturable (VBNC) State in Saccharomyces cerevisiae
Source: PLoS One. 2013 Oct 29;8(10):e77600. doi: 10.1371/journal.pone.0077600 (PMC3812164; doi:10.1371/journal.pone.0077600)

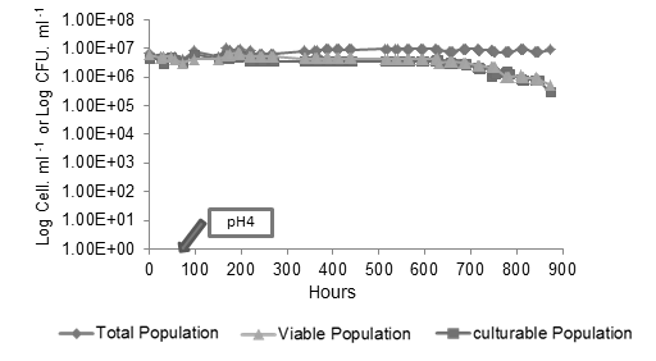

Supplement: Figure S1 — Effect of the increasing pH on the growth dynamic of a culture of S. cerevisiae S288C. Total cell counts (♦), culturable counts (▪), and viable counts (▴) are shown. pH increased at 3 days (72 h). The values presented are the average of three replicates of three separate experiments. (TIFF) [file pone.0077600.s001.tiff]

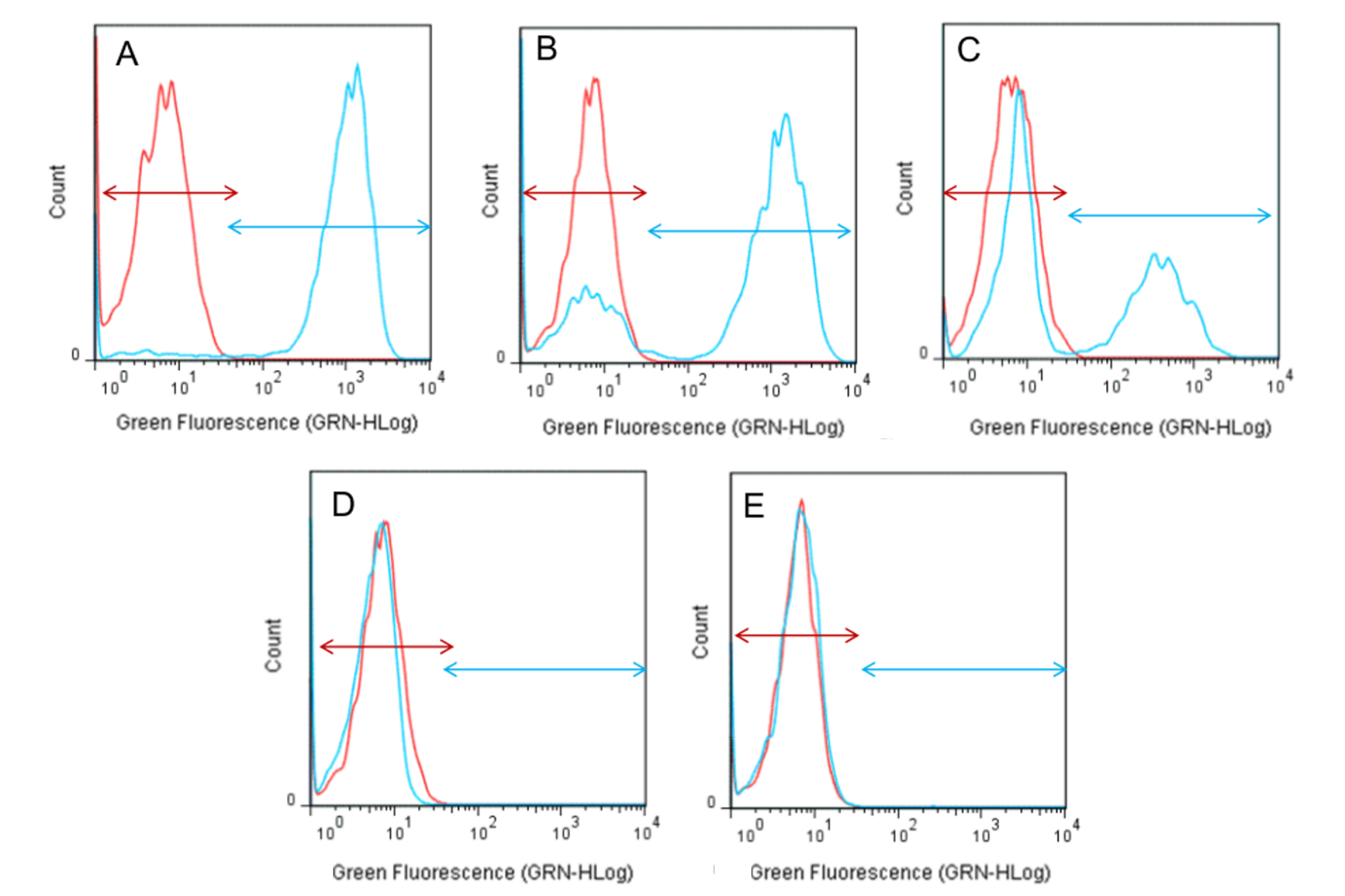

Supplement: Figure S2 — FCM histograms of S. cerevisiae S288C cells stained with FDA. The cells were incubated with 10 g/L SO2 in Synthetic wine at 28°C. After 15 (B); 30(C); 45(D) and 60(E) min, the cells were collected, and the cell Green fluorescence intensity was analyzed by FCM, Panel A represents control cells in the absence of SO2 (0 min). The Green fluorescence intensity (GRN-HLog) is represented on the x-axis, and cell counts are represented on the y-axis. Panels show the fluorescence of S. cerevisiae S288C before (red arrow; self-fluorescence) and after (blue arrow) staining with FDA. One representative experiment of the three performed is shown. (TIFF) [file pone.0077600.s002.tiff]
